# Tyrphostin AG1024 Suppresses Coronaviral Replication by Downregulating JAK1 via an IR/IGF-1R Independent Proteolysis Mediated by Ndfip1/2_NEDD4-like E3 Ligase Itch

**DOI:** 10.3390/ph15020241

**Published:** 2022-02-17

**Authors:** Cheng-Wei Yang, Yue-Zhi Lee, Hsing-Yu Hsu, Guan-Hao Zhao, Shiow-Ju Lee

**Affiliations:** Institute of Biotechnology and Pharmaceutical Research, National Health Research Institutes, Miaoli 35053, Taiwan; janeway@nhri.edu.tw (C.-W.Y.); biology213@nhri.org.tw (Y.-Z.L.); sandyshi720218@nhri.edu.tw (H.-Y.H.); 0921490201@nhri.edu.tw (G.-H.Z.)

**Keywords:** AG1024 (tyrphostin), coronavirus, E3 ligase, IGF-1R, Janus kinase, NEDD4, Ndfip1/2, proteolysis

## Abstract

JAK1 depletion or downregulation was previously reported to account for coronavirus inhibition. Here, we found that AG1024, an IR (insulin receptor) and IGF-1R (insulin-like growth factor 1 receptor) inhibitor, diminishes JAK1 protein levels and exerts anti-coronaviral activities with EC_50_ values of 5.2 ± 0.3 μM against transmissible gastroenteritis coronavirus (TGEV) and 4.3 ± 0.3 μM against human flu coronavirus OC43. However, although the IR and IGF-1R signaling pathways are activated by insulin or IGF-1 in swine testis cells, they are not triggered upon TGEV infection. AG1024, therefore, inhibits coronaviral replication and downregulates JAK1 protein levels independently of IR and IGF-1R. Moreover, JAK1 proteolysis caused by AG1024 was found through activation of upstream Ndfip1/2 and its effector NEDD4-like E3 ligase Itch. In addition, ouabain, which was reported to mediate JAK1 proteolysis causing anti-coronaviral activity by activation of Ndfip1/2 and NEDD4 E3 ligase, additively inhibited anti-coronaviral activity and JAK1 diminishment in combination with AG1024. This study provides novel insights into the pharmacological effects of AG1024 and Itch E3 ligase mediated JAK1 proteolysis and identified Ndfip1/2 as a cognate effector for JAK1 proteolysis via the diversified E3 ligases NEDD4 and NEDD4-like Itch. These findings are expected to provide valued information for the future development of anti-viral agents.

## 1. Introduction

The Janus kinase (JAK) family comprises four tyrosine kinases, TYK2, JAK1, JAK2, and JAK3, which share significant structural homology and initiate signaling transduction by associating with cytokine and hormone receptors [1,2]. JAK1 is the most promiscuous partner among the JAK family members forming the activated dimerized receptor complexes in which it pairs/partners with the other JAK members, and thus plays a vital role in most JAK family associated signaling pathways; in particular, it is essential for signaling certain type I and type II cytokines or interferons [1,3,4].

JAK1 degradation is associated with the regulation of its coupled biological functions, but only a few ubiquitination proteases and phosphatases, as well as the Ndfip1/2 activated proteolysis ligase NEDD4 (Neural Precursor Cell Expressed Developmentally Down-Regulated Protein 4; NEDD4 E3 Ubiquitin Protein Ligase), are involved in JAK1 degradation [5,6,7]. Previously, we discovered that JAK1 depletion or downregulation can account for coronavirus inhibition and that cardenolides down-regulate JAK1 in a Na^+^/K^+^-ATPase-independent manner by activation of NEDD4 family-interacting protein 1/2 (Ndfip1/2) and NEDD4 E3 ligase to mediate JAK1 proteolysis and degradation [8]. Therefore, activation of Ndfip1/2 by other means may constitute a novel route for JAK1 down-regulation.

The E3 ligase family comprises more than 600 E3 ligases grouped into three subfamilies and 28 members [9,10,11]. They catalyze protein ubiquitination, mediating diverse cellular biological functions [12]. However, whether NEDD4 E3 ligase is the only kind of E3 ligase associated with Ndfip1/2 and implicated in JAK1 degradation remains unknown.

AG1024 is a reversible, competitive inhibitor of insulin receptor (IR) and insulin-like growth factor 1 receptor (IGF-1R) [13] that induces apoptosis, imparts anti-cancer activity, and significantly delays tumor growth [14]. The inhibition of melanoma cell proliferation by AG1024 occurs partly through its acceleration of the degradation of phosphorylated forms of retinoblastoma protein by a mechanism involving protein degradation [15]. However, no anti-viral activities have been reported for AG1024.

The JAK signaling pathways activated through interferons is part of mammalian anti-viral defense [16,17,18,19,20,21]. Although viruses have evolved multiple mechanisms to inhibit IFN/JAK1 signaling to allow for increased viral replication and spread [17], some hijack the host cell’s JAKs/STATs to exert pleiotropic functions, including transformation and promiscuous transcriptional activation [20]. In addition to agents that block viral infectivity and replication through direct JAK1/2 inhibition [22], JAK1 protein levels downregulated by cardenolides via Ndfip1/2 and NEDD4 E3 ligase mediated proteolysis also contribute to coronavirus inhibition [8].

Herein, we report our discovery that AG1024 can inhibit coronaviruses and explore the underlying mechanism of action. Treatment of TGEV infected ST cells with AG1024 was found to activate a novel association of Ndfip1/2 with NEDD4-like E3 ligase Itch, and the subsequent JAK1 proteolysis/downregulation played a role in the IR/IGF-1R-independent anti-viral activity of AG1024.

Furthermore, the combined treatment of AG1024 and ouabain resulted in an additive inhibition of both anti-coronaviral activity and JAK1 diminishment via the activation of two different E3 ligases. This study provides novel insights into the pharmacological effects of AG1024, and the NEDD4-like Itch E3 ligase mediated JAK1 proteolysis and identified Ndfip1/2 as a cognate effector for JAK1 proteolysis via diversified E3 ligases, e.g., NEDD4 and NEDD4-like Itch. Ndfip1/2 and associated E3 ligases, therefore, merit further study pursuant to the development of anti-viral agents.

## 2. Results

### 2.1. Tyrphostin AG1024 Inhibited TGEV and HCoV-OC43 Coronaviral Activities

During our previous screening of inhibitors for the signaling pathways involved the anti-coronaviral activity of ouabain [8,23], we discovered that the cardenolide ouabain suppressed coronaviral replication via augmenting a Na^+^/K^+^-ATPase-dependent PI3K_PDK1 axis signaling, and that this effect could be antagonized by LY294002, a PI3K inhibitor [23]. In addition, we also discovered that ouabain also suppressed coronaviral replication by downregulating JAK1 mediated by Ndfip1/2 and NEDD4 E3 ligase via Na^+^/K^+^-ATPase-independent proteolysis and that tyrphostin AG1024 (an IR/IGF-1R inhibitor) but not picropodophyllin (PPP) (a selective IGF-1R inhibitor) significantly inhibited TGEV replication by ~84% (residual activity ~16 ± 6%) at a concentration of 10 μM, and enhanced the anti-TGEV activity of ouabain (Table 1).

**Figure 1:**
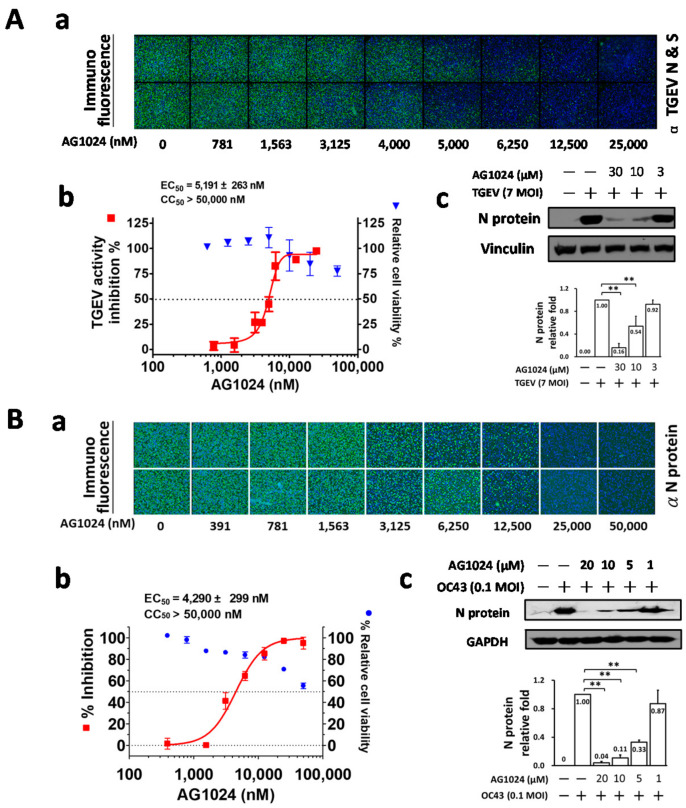
AG1024 exerted anti-coronaviral activity against TGEV and HCoV-OC43. (**A**) AG1024 inhibited TGEV coronaviral activity in a dose-dependent manner. IFA was performed to examine the dose responses of AG1024 on the viral inhibition in TGEV infected ST cells (**A**(**a**,**b**)). Western analysis with an antibody against TGEV N protein was also performed for an additional anti-viral inhibition measurement for AG1024 against TGEV (**A**(**c**)). Cell viability was determined by the CellTiter 96 AQueous Non-Radioactive Cell Proliferation Assay kit (MTS) after 6 h. (**B**) AG1024 inhibited HCoV-OC43 coronaviral activity in a dose-dependent manner. IFA were performed to examine the dose responses of AG1024 on the viral inhibition in HCoV-OC43 (0.1 MOI) infected HCT-8 cells after 24 h treatment (**B**(**a**,**b**)). Western analysis with antibody against HCoV-OC43 N protein was also performed for an additional viral inhibition measurement for AG1024 against HCoV-OC43 (0.1 MOI) in HCT-8 cells (**B**(**c**)). Hoechst dye was applied to counter-stain the nuclei (blue) and used to determine relative cell viability, which was used to normalize anti-viral activity. **, *p* < 0.01. Data shown are averages ± S.D. of three independent experiments.

Here, we further dissect the anti-viral activity of AG1024 against TGEV (Figure 1A) and HCoV-OC43 (Figure 1B) by IFA and western analysis. The EC_50_ values, determined by IFA, of AG1024 were found to be 5.2 ± 0.3 μM and 4.3 ± 0.3 μM for TGEV and HCoV-OC43 respectively (Figure 1A(a,b),B(a,b)).These results were further confirmed by western analyses with antibody against respective nucleocapsid (N) protein (Figure 1A(c),B(c)).

### 2.2. Coronaviral Inhibition of Tyrphostin AG1024 Is IR/IGF-1R Signaling Independent

**Figure 2:**
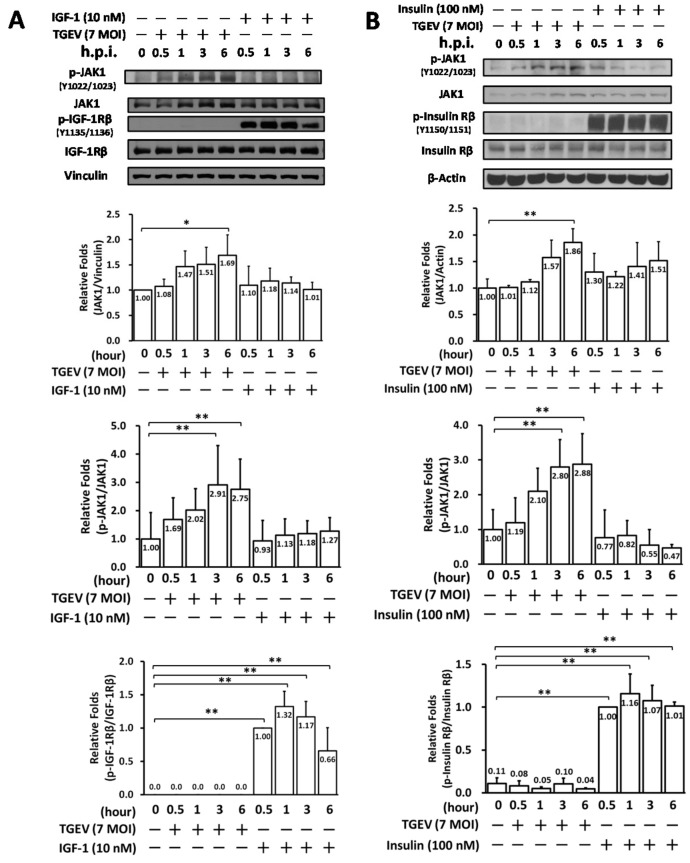
TGEV infection did not activate IR/IGF-1R signaling but induced JAK1 activation in the ST cell. ST cells were infected by TGEV at an MOI of 7 or respectively stimulated with IGF-1 (10 nM) (**A**) or insulin (100 nM) (**B**). The resulting cell lysates, harvested at the indicated time points of 0.5 h, 1 h, 3 h, and 6 h, were subjected to western analyses with antibodies against p-JAK1, JAK1, p-IGF-1R, IGF-1R, p-insulin R, insulin R or the internal loading controls vinculin or β-actin, respectively. *, *p* < 0.05; **, *p* < 0.01. Results shown are averages ± SD from three independent experiments.

Subsequently, we examined whether IR/IGF-1R signaling in ST cells occurs upon TGEV infection or insulin/IGF-1 stimulation. On the one hand, IR and IGF-1R activation/phosphorylation were dramatically induced upon adding IGF-1 (Figure 2A) or insulin (Figure 2B). On the other hand, however, no significant phosphorylation of IR or IGF-1R was found upon coronaviral infection of ST cells for 6 h, even though JAK1 activity (Figure 2A,B) and viral replication were increased, the latter indicated by N protein expression levels (Figure 1A). Since IR and IGF-1R activations/phosphorylations did not occur in coronaviral infected ST cells, thus we concluded that coronaviral inhibition by tyrphostin AG1024 (Figure 1) occurs independently of IR/IGF-1R signaling (Figure 2).

### 2.3. AG1024 Treatment Resulted in JAK1 Diminishment/Downregulation and Inhibited Viral N Protein Expression in TGEV Infected ST Cells

**Figure 3:**
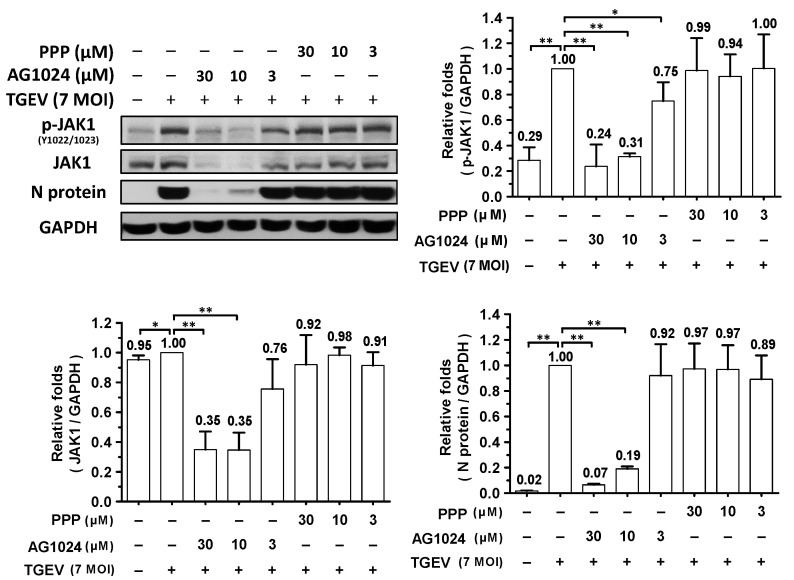
AG1024 diminished TGEV induced JAK1 activation/phosphorylation and inhibited TGEV activity in a dose-dependent manner. ST cells were pretreated with various concentrations of AG1024 or PPP respectively for 1 h prior to infection by TGEV at an MOI of 7. The resulting cells were harvested at 6 h.p.i. and then subjected to western analyses with antibodies against p-JAK1, JAK1, N, or an internal loading control GAPDH, respectively. *, *p* < 0.05; **, *p* < 0.01. Results shown are averages ± SD from three independent experiments.

Levels of both regular and phosphorylated JAK1 significantly increased in ST cells upon their infection with TGEV, but not after treatment with IGF-1 or insulin (Figure 2). Previously, we discovered that JAK1 depletion or downregulation can account for coronavirus inhibition and that a reduction in JAK1 levels contributes to the anti-viral activity of ouabain in a Na^+^/K^+^-ATPase-independent manner [8], so here we also examined the effect of AG1024 on JAK1 protein levels in TGEV infected ST cells. AG1024 was found to significantly reduce JAK1 protein and phosphorylation levels and viral replication (indicated by a decrease in N protein expression levels) in a dose-dependent manner (Figure 3). Nonetheless, the selective IGF-1R inhibitor PPP did not significantly inhibit TGEV activity (based on N protein expression levels) or reduce JAK1 levels (Figure 3). The mechanisms underlying the induction of JAK1 diminishment by AG1024 were studied as follows.

### 2.4. AG1024 Diminished JAK1 Protein Levels Mainly through Proteolysis

**Figure 4:**
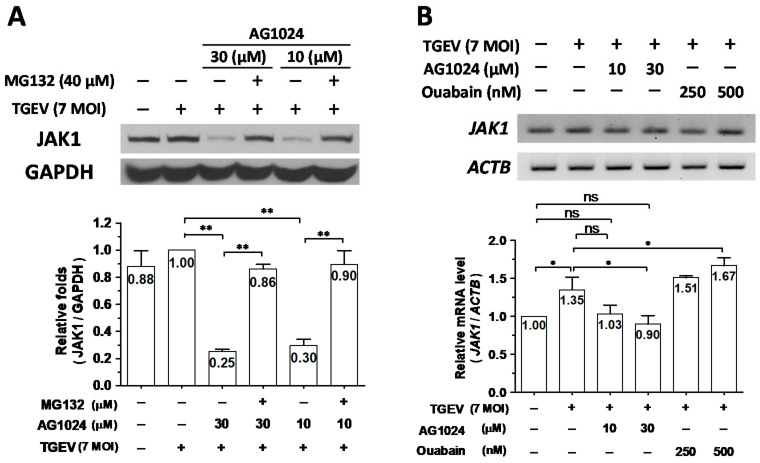
AG1024 diminished JAK1 protein levels mainly through proteolysis. (**A**) Proteasome inhibitor MG132 significantly restored AG1024 diminished JAK1 protein level. (**B**) The effect of AG1024 treatment on the JAK1 transcriptional activity as determined by RT-PCR. ST cells were treated with AG1024, MG132, or ouabain as indicated and harvested at 6 h.p.i. of TGEV (MOI:7). The resulting lysates were subjected to western analyses (**A**) or semi-quantitative RT-PCR (**B**), respectively. *, *p* < 0.05; **, *p* < 0.01. Results shown are averages ± SD from three independent experiments.

Previously, we reported that ouabain downregulates JAK1 protein levels through a proteolysis mediated by activating the Ndfip1/2_NEDD4 E3 ligase cascade [8]. Therefore, we wondered whether this mechanism could also account for AG1024 induced JAK1 proteolysis. In TGEV-infected and AG1024 (30 M and 10 M) treated ST cells, JAK1 protein levels were decreased to about 25–30% of their levels compared to TGEV-infected but AG1024-untreated cells, a decrease of about 70–75%. After TGEV infection, AG1024-treated cells further treated with the proteasome inhibitor MG132 restored JAK1 protein levels from ~25–30% back to ~90% (Figure 4A). On the other hand, whereas JAK1 transcription increased by ~35% in ST cells upon TGEV infection compared to uninfected control (Figure 4B), JAK1 protein levels only increased by about ~10% (Figure 4A), and AG1024 treatment reduced transcription from ~135% back to ~100%, the level of uninfected control (Figure 4B).

Based on these results, we concluded that AG1024 diminished JAK1 protein levels mainly through proteolysis (Figure 4). We next studied the underlying mechanisms of action of this effect.

### 2.5. Downregulation of JAK1 by AG1024 Induced Proteolysis Was Associated with Ndfip1/2

**Figure 5:**
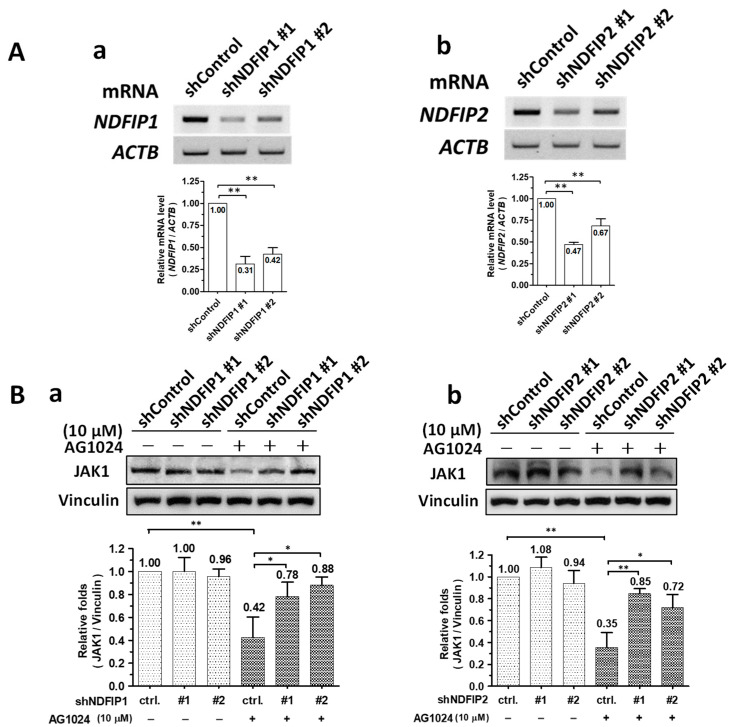
Ndfip1/2 were associated with AG1024 induced JAK1 diminishment. (**A**) The mRNA expression of either Ndfip1 (**a**) or Ndfip2 (**b**) were significantly depleted by gene silencing. The relative mRNA expression levels of Ndfip1 (**a**) or Ndfip2 (**b**) in ST cells harboring the respective Ndfip1-shRNA, Ndfip2-shRNA, or control shRNA are shown along with their respective quantitation as measured using semi-RT-PCR. (**B**) Depleting either Ndfip1 (**a**) or Ndfip2 (**b**) prevented the downregulation of the JAK1 protein level by AG1024 treatment. *, *p* < 0.05; **, *p* < 0.01. Results shown are averages ± SD from three independent experiments.

When Ndfip1 (Figure 5A(a)) or Ndfip2 (Figure 5A(b)) were knocked down (depleted) in ST cells by gene silencing to residual levels of ~30–40% and ~50–70%, respectively, JAK1 protein levels were not affected compared to parental sh-control cells (Figure 5B(a) left group & Figure 5B(b) left group). However, in the presence of AG1024, JAK1 protein levels in parental sh-control cells significantly decreased to about 40% of their initial value, a decrease of ~60% (Figure 5B(a,b)). In Ndfip1 (Figure 5B(a)) or Ndfip2 (Figure 5B(b)) depleted cells, JAK1 levels resisted AG1024 treatment, decreasing to ~70–90% of their initial value, corresponding to decreases of only about 10–20% in Ndfip1 depleted cells (Figure 5B(a)) and ~10–30% in Ndfip2 depleted cells (Figure 5B(b)). Therefore, the downregulation of JAK1 by AG1024 induced proteolysis was concluded to be associated with Ndfip1/2.

### 2.6. Downregulation of JAK1 by AG1024 Induced Proteolysis Was Mediated by NEDD4-like E3 Ligase Itch

**Figure 6:**
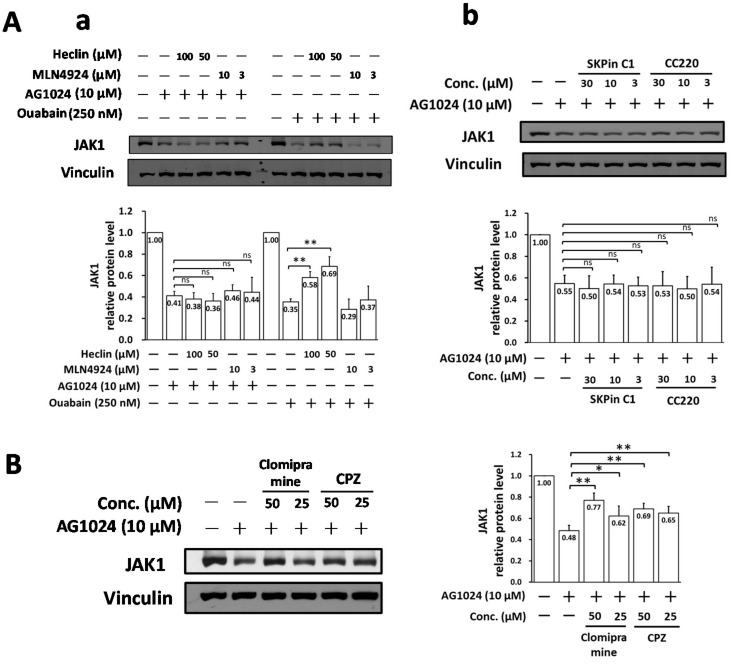
NEDD4-like Itch E3 inhibition restored the AG1024 downregulated JAK1. (**A**) Neither the NEDD4 inhibitor heclin (**a**) nor the skp2 inhibitors SKPin C1 and CC220 (**b**) restored AG1024 downregulated JAK1, whereas the NEDD4 inhibitor heclin restored ouabain diminished JAK1 protein level. (**B**) Itch E3 inhibitors clomipramine and CPZ significantly restored the AG1024 downregulated JAK1. TGEV infected (MOI: 7) ST cells were treated with ouabain, AG1024, heclin, MLN4924, SKPin C1, CC220, clomipramine, or CPZ as indicated and harvested at 5 h.p.i. MLN4924, a NEDD8 inhibitor, served as a non-specific reference control, and the concentrations were used as reported [24]. The concentrations of SKPin C1, CC220, clomipramine, or CPZ used herein were based on previous reports [25,26,27,28,29]. The resulting lysates were subjected to western analyses. *, *p* < 0.05; **, *p* < 0.01. Results shown are averages ± SD from three independent experiments.

We next examined whether the NEDD4 inhibitor heclin could also restore JAK1 levels downregulated by AG1024 in a dose-dependent manner, as is the case for ouabain [8]. As expected, we found that heclin did restore JAK1 levels downregulated by ouabain, but not those downregulated by AG1024, Figure 6A(a).

Other E3 ligases were subsequently examined. Clomipramine and CPZ, both inhibitors of Itch E3 ligase, significantly restored AG1024 downregulated JAK1 protein levels. We found clomipramine and CPZ restored JAK1 levels downregulated by AG1024 from ~48% back to 62–77% (an increase of ~14–29%) and 65–69% (an increase of ~17–21%), respectively (Figure 6B), whereas the skp2-type E3 inhibitors SKPin C1 and CC220 did not (Figure 6A(b)). Therefore, AG1024 downregulation of JAK1 proceeds at least partly through an IR/IGF-1R independent proteolysis mediated by a Ndfip1/2-associated NEDD4-like Itch E3 ligase cascade.

### 2.7. Combined Treatment of AG1024 and Ouabain Had an Additive Effect on Both Anti-Viral Activity and JAK1 Downregulation

**Figure 7:**
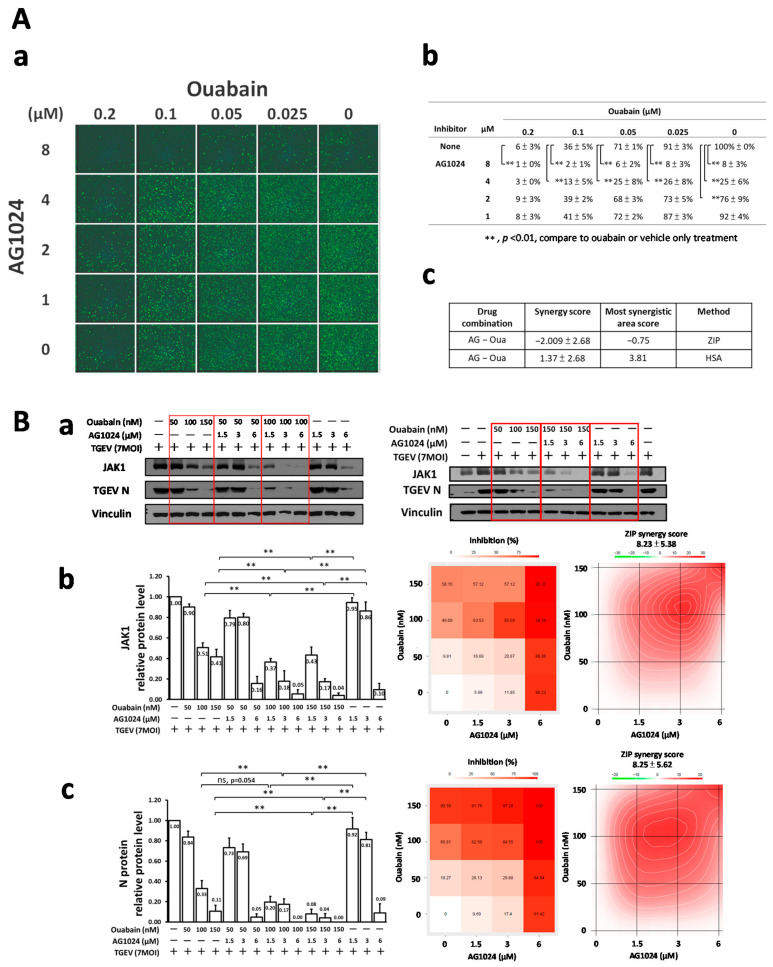
Additive effect of AG1024 and ouabain in anti-TGEV activity and JAK1-diminishment. (**A**) Combined treatment of AG1024 and ouabain profoundly and additively reduced TGEV infection as assayed by IFA in ST cells. (**B**) Combined treatments of AG1024 and ouabain additively diminish JAK1 protein levels. TGEV (MOI:7) infected ST cells were treated with compounds as indicated, and the resulting cells were subjected to IFA or harvested at 6 h.p.i. for subsequent western analyses. The results obtained from IFA (**A**(**a**,**b**)) or western **B**(**a**) were further analyzed by SynergyFinder(**A**(**c**),**B**(**b**,**c**)). **, *p* < 0.01. Results shown are averages ± SD from three independent experiments.

To dissect whether the two differential E3 ligase JAK1 proteolysis cascades mediated by Ndfip1/2 could work in concert with each other, anti-viral activity and JAK1 levels were examined by IFA and western analysis, respectively. The results (Figure 7A(a,b),B) demonstrated that ouabain significantly enhanced the anti-viral activities of AG1024 as examined by IFA (Figure 7A(a,b)) in a dose-matrix response experiment with antibodies against N and spike (S) protein of TGEV and by western analysis for JAK1 protein levels (Figure 7B). In addition, the abovementioned IFA and western results were subjected to a SynergyFinder analysis, which showed that combined treatments of AG1024 and ouabain were additive for anti-viral activity and JAK1 downregulation (Figure 7A(c),B(b,c)).

## 3. Discussions

JAK1 is the most promiscuous partner for the other JAK members. Therefore, it plays an essential role in JAK family associated signaling events, including various immune responses. Deletion (or loss) of JAK1 could cause an adverse effect in immune response. Loss of JAK1 in natural killer cells dramatically results in the reduction of natural killer cell numbers and causes the impaired development of bone marrow [30]. While depletion of JAK1 via E3 ligase associated degradation only blocks the particular associated JAK1 signaling, not comprehensive JAK1 inhibition, we should still be cautious for the risk in unexpected immune response or other advert effects.

**Figure 8:**
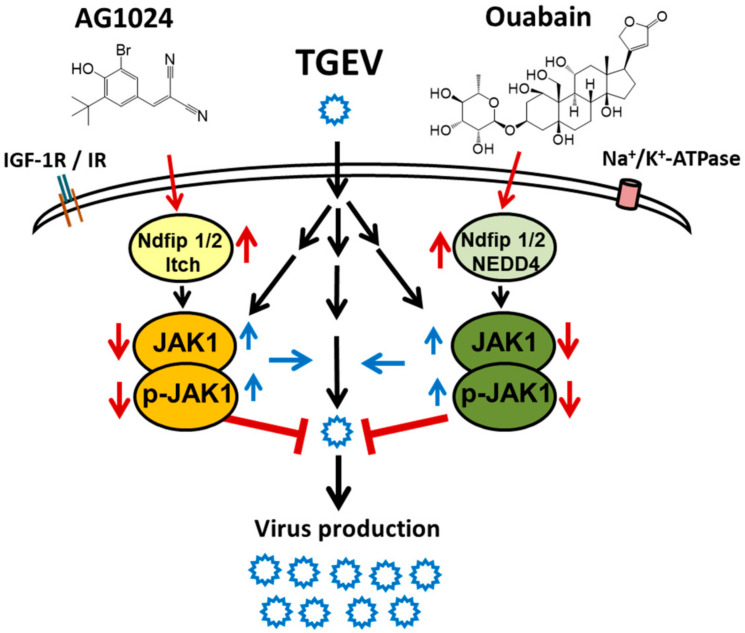
Summary scheme for the cellular effects of AG1024, and in combination with ouabain, on JAK1 degradation and coronaviral inhibition.

The ubiquitin E3 ligase family is large and diverse [9,10,11]. We previously reported that ouabain triggers the signaling event that leads NEDD4 E3 ligase to complex with Ndfip1/2 to recruit and proteolyze JAK1 [8]. Here, we report a complex of Ndfip1/2 and NEDD4-like Itch E3 ligase that also mediates JAK1 proteolysis upon tyrphostin AG1024 treatment. Because both ouabain and AG1024 inhibit coronaviruses through downregulation of JAK1 independently of their cognate receptors on the host cell membrane, Na^+^/K^+^-ATPase, and IR/IGF-1R, respectively. Therefore, they should have their own alternative targets that account for JAK1 downregulation. These alternative targets are presumably associated with JAK1 that pairs with a different JAK partner as in a wide variety of cytokine and hormone receptors [1,2,3,4] (Figure 8).

In addition, perturbation of cellular protein degradation using proteolysis-targeting chimeric molecules (PROTACs) is an emerging strategy in drug discovery [31]. PROTACs are heterobifunctional small molecules comprising a ligand for recruiting an E3 ligase located at one end of the molecule, a linker in the middle, and another ligand to bind the protein targeted for degradation at the other end. Whether AG1024 or cardenolides (e.g., ouabain) simultaneously interact with Ndfip1/2_E3 ligase and JAK1 like the PROTACs or through associated complex coupled members remains to be studied.

Moreover, since Ndfip1/2 was found to be associated with different E3 ligase members of NEDD4 or NEDD4-like Itch for JAK1 proteolysis, it would be an alternative drug target for JAK1 inhibition—activating Ndfip1/2 by overexpression or other approaches to downregulate JAK1 and its associated biological functions to treat disease. In addition, it remains to be explored whether there are complexes of Ndfip1/2 with E3 ligases other than NEDD4-like Itch and NEDD4 that are capable of JAK1 deregulation.

Our finding that the combined treatment of AG1024 and ouabain (Figure 7 and Figure 8) causes additive effects in anti-viral activity and JAK1 diminishment provides additional evidence that either Ndfip1/2_NEDD4 E3 ligase or Ndfip1/2_NEDD4-like Itch E3 ligase could account for the proteolysis of JAK1, and their downregulation of the JAK1 protein level arises from two different cognate upstream signaling pathways. One of these is activated by ouabain in a Na^+^/K^+^-ATPase-independent manner, and the other by AG1024, via an IR/IGF-1R independent signaling. Thus, the regulation of JAK1 proteolysis by which E3 ligase is suggested to arise from the cognate signaling complex partnered with JAK1.

The effects of AG1024 and ouabain on JAK1 protein levels and the associated anti-coronaviral activities are also additives (Figure 7 and Figure 8), confirming that the pathways by which these effects occur are independent and can work in concert as a regimen for viral inhibition. Therefore, the targeting of a selective molecular proteolysis, e.g., JAK1 proteolysis, is a valid approach for drug discovery and the development of anti-viral agents complementary to the targeting of enzymatic or biological functional inhibition. Our study also has yielded novel insight into the pharmacological effect of AG1024 in activation of Ndfip1/2_NEDD-4 like Itch E3 ligase, which may constitute the basis for the development of future anti-viral agents and PROTACs.

## 4. Materials and Methods

### 4.1. Cells, Viruses, Immunofluorescence Assay (IFA), and Cytotoxicity Assays

The resources, maintenance, passages used, and assurance of cell line-specific characteristics of Swine testicular (ST) epithelial cells (ATCC^®^CRL-1746™) and human colon adenocarcinoma cell line, HCT-8 (ATCC^®^ CCL-244™) were as described [8,32]. The maintenance and propagation of TGEV, as well as immunofluorescent assay (IFA) with antibodies against S and N proteins regarding TGEV infection, were also as described [8]. A multiplicity of infection (MOI) of 7 was used for TGEV to infect ST cells for all experiments. Cytotoxicity assays of TGEV infected ST cells with or without treatment of compounds were also carried out as described [8,33]. HCT-8 cells were cultured and maintained in the presence of 10% fetal bovine serum (FBS), while 2% FBS was used for compound treatment studies. HCT-8 cells were pretreated with compounds for 1 h prior to HCoV-OC43 infection (MOI: 0.1). The resultant cells at 24 h.p.i. were subjected to IFA 24 h.p.i. as described or harvested for western analyses using an antibody (MAB9013) against OC43 N protein [8,34].

### 4.2. Chemicals and Antibodies for Western Blot Analyses

DMSO (≧99.5%), human insulin (I2643, ≧98%, HPLC), ouabain (O3125, ≧95%, HPLC), and clomipramine hydrochloride (C7291, ≧98%, HPLC) were purchased from Sigma-Aldrich (St. Louis, MO, USA); MG132 (474790, ≧98%, HPLC) from Merck Millipore Calbiochem (Merck, La Jolla, CA, USA); AG1024 (S1234, ≧99.7%, HPLC), picropodophyllin (PPP, S7668, ≧99.5%, HPLC), MLN4924 (S7109, ≧99%, HPLC), chlorpromazine (CPZ, S5749, ≧97%, HPLC), Skp2 inhibitor C1 (SKPin C1, S8652, ≧99%, HPLC) and iberdomide (CC-220, S8760, ≧98%, HPLC) from Selleckchem (Houston, TX, USA); heclin (5433, ≧98%, HPLC) from Tocris Bioscience (Minneapolis, MN, USA); and human IGF-1 (CYT-216, ≧98%, SDS-PAGE) from ProSpec (Rehovot, Israel).

Western blotting was performed as described [35,36] with antibodies against β-actin (1:2000, catalog # 4970), glyceraldehyde 3-phosphate dehydrogenase (GAPDH) (1:2000, catalog # 2118), p-IGF-I Rβ (Tyr1135/1136)/insulin Rβ (Tyr1150/1151) (1:1000, catalog # 3024), IGF-I Rβ (1:1000, catalog # 3027), insulin Rβ (1:1000, catalog # 3025), p-JAK1 (Y1022/1023) (1:1000, catalog # 3331), and JAK1 (1:1000, catalog # 3344) from Cell Signaling Technology, Danvers, MA, USA; vinculin (GTX109749, 1:2000) (GeneTex, Irvine, CA, USA); OC43 N protein (MAB9013, 1:1000) (Merck, La Jolla, CA, USA) and an antibody against TGEV N protein, as described [35]. All these antibodies were used to detect their respective counterparts in ST cells as in previous reports [8,23,37]. Horseradish peroxidase-conjugated secondary antibodies (PerkinElmer, Inc., Waltham, MA, USA) and enhanced chemiluminescence detection reagents (Western Blot Chemiluminescence Reagent Plus; PerkinElmer, Inc., Waltham, MA, USA) were used according to the manufacturers’ instructions to detect antigen/antibody complexes. Vinculin, GAPDH, and β-actin were used as internal loading controls for western analyses.

### 4.3. RNA Isolation, Semi-Quantitative Reverse-Transcriptase Polymerase Chain Reaction (Semi-RT-PCR)

These experiments were performed as described [8]. Total RNA was extracted from the test cell lysates with TRIzol reagent (Invitrogen, Waltham, MA, USA) prior to RT-qPCR analyses. The primers used to amplify the PCR products of JAK1, β-actin, Ndfip1, and Ndfip2 are as follows: 5′-GTATGGCGGCATTCTCCAAA-3′ and 5′-TACTGCCCCTGAGCAAAGAG-3′ for JAK1; 5′-GGCTCAGAGCAAGAGAGGTATCC-3′ and 5′-GGTCTCAAACATGATCTGAGTCATCT-3′ for β-actin; 5′-GCAATCAAGTCTGTGATGTA-3′ and 5′-GCCAGCATTATACATATCTTAC-3′ for Ndfip1; 5′-CGGGCAGGATGGATCATCAC-3′ and 5′-GTACTTCCACGGTAATACTA-3′ for Ndfip2. The following conditions were used to amplify the targeted cDNAs: 30 cycles of 95 °C for 15 s, 55 °C for 15 s, and 72 °C for 30 s. Electrophoresis on 2% agarose gel containing ethidium bromide along with DNA markers was performed to resolve the final PCR products. The acquisition of relative PCR product amounts was accomplished using the ImageJ Analyzer program software (version 15.2a).

### 4.4. Gene Silence

ST cells harboring Ndfip1 shRNA #1, Ndfip1 shRNA #2 (clone IDs: #1 TRCN0000276095; #2 TRCN0000276161), Ndfip2 shRNA #1, Ndfip2 shRNA #2 (clone IDs: #1 TRCN0000365568; #2TRCN0000370776) or negative control-shRNA #1, negative control-shRNA #2 (shLacZ, clone IDs: #1 TRCN0000231722-TRC05; #2 TRCN0000072224-TRC01) (Academia Sinica, Taipei, Taiwan) for respective gene silencing were prepared as described previously [38]. After validation of knockdown expression of Ndfip1 or Ndfip2, the cells were used for the followed experiments.

### 4.5. Drug Combination Study

Viral inhibition by AG1024, ouabain, or in their combination as measured by IFA or western analysis was assessed using a drug dose-response matrix. The online tool SynergyFinder (https://synergyfinder.fimm.fi/, accessed on 26 October 2021) was applied to the analysis of the results from three independent experiments to obtain the average synergy scores. ZIP or HSA synergy scores were calculated and plotted. The interaction between two drugs was considered synergistic for ZIP and HSA synergy scores greater than 10; to be additional for scores between −10 and 10; and to be antagonistic for scores of less than −10.

### 4.6. Statistical Analysis

The two-tailed unpaired Student’s *t*-test was used to evaluate the statistical significances in the dose-effect of drug treatments. * and ** denote statistical significances of *p* < 0.05, and *p* < 0.01 respectively.

## 5. Conclusions

Tyrphostin AG1024 suppresses coronaviral replication by downregulating JAK1 via an IR/IGF-1R independent proteolysis mediated by Ndfip1/2_NEDD4 like E3 ligase Itch. In addition, Ndfip1/2 may be a common effector for JAK1 proteolysis via diversified E3 ligases, recruited and destinated by the upstream JAK1 partnered signaling.

## Figures and Tables

**Table 1:** Inhibition of TGEV activity by AG1024 and ouabain, respectively, or both in combination. Shown are residual activities after the combined treatment of AG1024 or PPP with ouabain compared to DMSO vehicle treatment, the TGEV activity of which was defined to be 100%. IFA, as described in the Methods section, which used antibodies against viral nucleocapsid (N) and spike (S) protein to detect their expressions, was applied to measure TGEV activity.

		Ouabain (µM)			
Inhibitor	µM	0.3	0.2	0.1	0
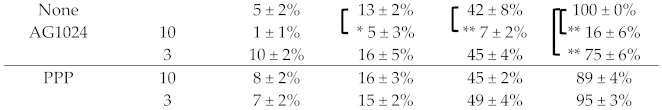					

Results shown are averages± SD from three independent experiments, each conducted in duplicate. *, *p* < 0.05; **, *p* < 0.01, compare to ouabain or vehicle only treatment.

## Data Availability

Data is available within the article.

## Abbreviations

JAK1: Janus kinase 1; IR: insulin receptor; IGF-1R: insulin-like growth factor 1 receptor; NEDD4: neural precursor cell expressed developmentally down-regulated protein 4; Ndfip1/2: NEDD4 family interacting protein 1/2; IFN: interferon; STAT: signal transducer and activator of transcription; TGEV: transmissible gastroenteritis coronavirus; PI3K: phosphatidylinositol 3-kinase; PDK1: 3-phosphoinositide-dependent kinase 1; PPP: picropodophyllin; HCoV-OC43: human flu coronavirus OC43; IFA: immunofluorescence assay; ST cells: swine testicular cell; N protein: nucleocapsid protein; S protein: spike protein; CPZ: chlorpromazine; skp2-type E3: S-phase kinase-associated protein 2-type E3 ubiquitin ligase; MOI: multiplicity of infection.
